# Factor analysis of the Clinical Outcomes in Routine Evaluation – Outcome Measures (CORE-OM) in a Kenyan sample

**DOI:** 10.1186/s40359-018-0260-1

**Published:** 2018-10-01

**Authors:** Fredrik Falkenström, Manasi Kumar, Aiysha Zahid, Mary Kuria, Caleb Othieno

**Affiliations:** 1 Department of Behavioural Sciences and Learning, Linköping University, SE-581 83 Linköping, Sweden; 2 Department of Psychiatry, University of Nairobi, P.O. Box 19676, Nairobi, 00202 Kenya; 3 Honorary Research Fellow, Research Dept of Clinical Health and Educational Psychology, University College London, London, WC1E 7BT UK; 4 Queen Mary’s College, Miles End, London, E1 4NS UK

**Keywords:** Psychological assessment, Outcome measurement, Psychotherapy, Factor analysis, Psychological distress

## Abstract

**Background:**

There is no generic psychotherapy outcome measure validated for Kenyan populations. The objective of this study was to test the acceptability and factor structure of the Clinical Outcomes in Routine Evaluation – Outcome Measure in patients attending psychiatric clinics at two state-owned hospitals in Nairobi.

**Methods:**

Three hundred and forty-five patients filled out the CORE-OM after their initial therapy session. Confirmatory and Exploratory Factor Analysis (CFA/EFA) were used to study the factor structure of the CORE-OM.

**Results:**

The English version of the CORE-OM seemed acceptable and understandable to psychiatric patients seeking treatment at the state-owned hospitals in Nairobi. Factor analyses showed that a model with a general distress factor, a risk factor, and a method factor for positively framed items fit the data best according to both CFA and EFA analysis. Coefficient Omega Hierarchical showed that the general distress factor was reliably measured even if differential responding to positively framed items was regarded as error variance.

**Conclusions:**

The English language version of the CORE-OM can be used with psychiatric patients attending psychiatric treatment in Nairobi. The factor structure was more or less the same as has been shown in previous studies. The most important limitation is the relatively small sample size.

## Background

Colonialism had a debilitating impact on the expression of psychological distress in the Kenyan people. Most psychiatric and public health facilities during colonial rule (Kenya got independence only around 1963) were earmarked for Europeans, followed by Indians who were brought to serve in colonial administration, and native Kenyans were neglected with limited care or consideration of their distress [1]. To this day, Kenyan people visit psychiatric hospitals or seek services only when they are in tremendous adversity where either their livelihood or everyday functioning is severely impacted. The notions of well-being beyond this reality, including subjective well-being and improved quality of life, have not been promoted in the general public consciousness.

In 2011 the Kenya National Commission on Human Rights (KNCHR) conducted a human rights-focused audit of the mental health system. They concluded that “as a result of stigma and discrimination against mental illness and persons with mental disorder, the policies and practices of the Government of Kenya have been inadequate and resulted in a mental health system that is woefully under-resourced and unable to offer quality inpatient and outpatient care to the majority of Kenyans who need it” (p. iii, [2]). This devastating conclusion shows the great need for developing mental health treatments for the Kenyan population. One step in this direction is to start using psychometrically sound instruments for tracking the course of psychological problems, well-being, and functioning of patients undergoing psychological and psychiatric treatments.

The Clinical Outcomes in Routine Evaluation – Outcome Measure (CORE-OM; [3]) was developed to be a broad measure of psychological distress that could be used for assessing change in psychotherapy in clinical settings.^1^ The CORE-OM is widely used in the United Kingdom [4, 5], has been used in psychotherapy studies for measuring outcome [6] and has been translated into several languages. The items cover four domains: well-being (4 items), problems (12 items), functioning (12 items) and risk to self and to others (6 items). Items were developed to be sensitive at different severity levels. Several factor analytic evaluations of the CORE-OM have been reported. The initial Principal Component Analysis reported by the test developers [3] suggested three components; a first component that explained a large amount of variance (38%), plus a risk component and a positively worded component.

A later Confirmatory Factor Analysis, also by the test developers [7], suggested that a bifactor model with a “g-factor” plus method factors (positive/negative responding) and a risk to self and others factor, explained most of the variation in observed item responses. Although their best fitting model included the well-being, psychological problems and functioning domains, factor loadings for these subscales were so small that they did not explain much variance in items.

Factor analysis of the Norwegian version of the CORE-OM [8] also suggested a bifactor model. However, in this version the method factors did not contribute to model fit. The best fitting model was a bifactor model with a general distress factor and the four CORE-OM domains. A difference in the modeling approach compared to the British ones [3, 7] was that the Norwegian authors [8] treated the CORE-OM response scale as ordinal, while the British ones treated it as continuous.

Another research group working with the English version of the CORE-OM suggested an Item Response Theory approach called Mokken scaling to explain the CORE-OM item responses [9]. Specifically, Mokken scaling assumes unidimensionality, i.e. one latent factor, but items are differentially “difficult” in the sense that different items provide information at different levels of the latent factor. So, rather than items grouping into different subscales that provide information about different types of psychological problems/well-being (as in factor analysis), all items load on a general psychological distress factor but some items differentiate among more severe levels of distress while others differentiate better among less severe levels of distress. Using such an approach, the authors found that the well-being items tended to inform about the lower levels of distress while the risk to self and others items informed about the highest levels of distress (with other items in-between). That approach also suggested that the CORE-OM could be substantially shortened, with suggestions that around 6–8 items would be enough.

The CORE-OM has been translated and psychometrically evaluated in several languages, e.g. Swedish [10], Norwegian [8], Italian [11], Icelandic [12] and Spanish [13].^2^ We know of only one African evaluation of the CORE-OM, and that is from South Africa [14]. As in the present study, the South African evaluation used the English language version of the CORE-OM but evaluated it in for use in the African cultural context. This is a slightly different issue from evaluating a translation, in the sense that it is not the issue of translation that needs to be evaluated but only the application in a different culture. If the existing measure seems to work in these new cultures, that is an advantage since no adaptations need to be done. If not, the instrument needs to be changed.

The purpose of the present study was to test the of the CORE-OM factor structure in a Kenyan sample. We wanted to test whether previous factor analytic results held up in our data, and if the evidence was to the contrary to explore what alternative structure might fit better.

## Methods

### Participants

Three hundred and forty-five participants were recruited. The participants either attended one of four clinics; Youth clinic (*n* = 140), Department of Mental Health (*n* = 14), Psychiatric Clinic (*n* = 11) and Mathare Hospital (*n* = 180). The participants’ ages ranged from 18 to 60 years old (*M* = 28.9, *SD* = 9.8). The majority of the participants were male (72.6%), while 27.4% of the sample were female, the remaining two participants did not indicate their gender. Patients attended between one and eight sessions of treatment (M = 2.7); the present study uses only baseline data. The most common disorders that patients were seeking treatment for were alcohol or drug addictions (54.8%), psychosis (17.5%), depression (16.9%) and anxiety/stress (12.0%). Other identified problems that were less common included interpersonal problems, physical problems, work/academic problems, self-esteem problems, trauma/abuse, etc. The patients were treated with a variety of medications and therapies, for example, Cognitive Behavioral Therapy, Interpersonal Psychotherapy, Addiction Counseling, Supportive Therapy, Family Therapy, Psychoeducation and Brief Solution Focused Therapy. All participants of our study were out-patients implying that they had recovered enough to resume some degree of normal functioning and if they first came to Mathare hospital due to a legal proceeding; they were deemed safe and mentally stable to be integrated with the society. The study received ethical approval (number P85/02/2014) from KNH/UoN Ethics & Research Committee (KNH/UoN-ERC) and took written informed consent was obtained from all study participants.

### Measures

The Clinical Outcomes in Routine Evaluation – Outcome Measure [3] consists of 34 items about how the patient has been feeling over the past week in four particular domains; well-being (4 items; e.g. “I have felt O.K. about myself”), problems (12 items; e.g. “I have been disturbed by unwanted thoughts and feelings”), functioning (12 items; e.g. “I have felt warmth or affection for someone”) and risk (6 items: e.g. “I have threatened or intimidated another person”). Each item of the CORE-OM is rated on a Likert scale ranging from 0 to 4 (0 = not at all, 4 = most of the time). Eight of the items (24%) are positively framed. Higher scores indicate greater levels of distress. Prior research has established acceptability, internal consistency, test-retest reliability, convergent validity, differentiation between clinical and non-clinical samples and sensitivity to change [3].

### Procedure

Most of the participants were recruited from Kenyatta National Hospital (KNH) clinics. KNH is a large general hospital with 1500 bed capacity. It also runs outpatient clinics in various disciplines such as medical, psychiatric, and surgical clinics. In addition, there is a psychiatric department that offers counseling and psychotherapy services to patients referred from within and outside the hospital. The Patient support Centre located within KNH started off as a service for patients diagnosed with HIV and other medical problems that needed psychological support. Currently wider ranges of patients attend the Centre including those with purely psychological or social support. The study participants were recruited from two of these clinics; Clinic 24 and the Patient Support Centre. The psychiatric outpatient clinic runs once a week on Wednesday morning and roughly 10 new patients are seen each week. A similar number of new patients are seen at the PSC each week.

Mathare Hospital is a national psychiatric teaching and referral hospital. It was established in 1911 during British Colonial rule and is situated about 10 km from the centre of Nairobi (Kenya’s capital city) and about 14 km from Kenyatta National Hospital. The hospital now has over 650 beds, for both male and female patients and it has a drug rehabilitation centre, inpatient care for prisoners, a child and adolescent outpatient clinic amongst its prominent clinics. It has over a dozen Government-employed psychiatrists with several technicians, pathologists, nurses and health workers affiliated with the hospital. The institution has a long history of stigmatization and usually its clientele include those who cannot afford private services and are considered too disturbed to be managed in any other private or public facility, or in the community. Whilst its primary catchment is Nairobi it does have patients from rural Kenyan towns.

Data was collected from April 2014 to March 2015. After each therapy session, patients were asked by a research assistant to take about 5–10 min to fill in the CORE-OM questionnaire. Only the first session CORE-OM was used in the present study. No eligible participant declined to participate in our study. Despite this, due to time constraints on the research assistants, data could not be meticulously collected from all patients attending the clinics throughout the year. There were several reasons for this. At times these appointments were changed due to personal circumstances of the patients, at times due to financial constraints associated with finding travel or hospital fee and at other times there were overlapping appointments with other hospitals or hospital clinics that made it difficult to track participants consistently. The patient flow in the clinics varied depending on the time of the year making it difficult to predict who would come back on their scheduled visit. The research assistants were postgraduate students working part-time on the project. The data that was missing for this reason was most likely completely random. If this assumption is true, results would be unaffected by the missing data. Such practical barriers have been commonly noted in mental health services research in resource constraint settings.

### Statistical analysis

The CORE-OM data were first subjected to Confirmatory Factor Analysis (CFA) using models specified by theory and prior research. Since the originally specified model for the CORE-OM, with four correlated factors corresponding to the four domains, has been refuted by several factor analyses, we did not consider that model. The models compared were; 1) a bifactor model with a general distress factor plus the four CORE-OM domains, 2) a bifactor model with a general distress factor and a risk factor, 3) a bifactor model with a general distress factor, a method factor for positively keyed items, and the four CORE-OM domains, and 4) a bifactor model with a general distress factor, a method factor for positively keyed items, and a risk factor. Note that in contrast to prior CORE-OM factor analyses [7, 8] we did not estimate two separate method factors for positive and negative responding, respectively, since negative responding would not be possible to distinguish from the general distress factor and would thus be redundant. The positive responding factor loadings were constrained to 1, under the assumption that a method factor is likely to affect all items equally.

Since the data for these analyses were from a very different cultural context than the British data, we were prepared that data might not fit our models very well. In case models would fit poorly, we planned to use Exploratory Factor Analysis (EFA) to see whether another structure might be more appropriate for the Kenyan CORE-OM data. In addition to the use of model fit criteria, which tend to be hard for factor models with many indicators to achieve [15], we also evaluated the practical significance of our models using Explained Common Variance (ECV; [16]) which is a measure of “essential unidimensionality” that can be used as criterion for when a model with a strong G-factor is unidimensional enough to be used as such in practice. The ECV is defined as the amount of variance explained by the general factor divided by the total variance explained by all factors (general plus specific factors). Reliability of factors was determined using the Coefficient Omega Hierarchical. All analyses used the covariance matrix of the baseline CORE-OM measure, and were estimated with Maximum Likelihood estimation using Mplus 8, version 1.5 [17].

## Results

### Descriptive statistics

Item-level missing data was sparse, with at most four patients (1%) skipping some items. All items had skewness statistics between − 0.1 and 1.7, and kurtosis between − 1.3 and 1.7. Mean level of distress at intake (CORE-OM clinical score = average of all items × 10) was 14.8 (SD = 7.9, range 1.8–37.9).

### Confirmatory factor analysis

Table 1 shows model fit indices for the models tested. All models that allowed the four domains to be correlated yielded correlations > 1.0 between Well-being and Problems, indicating that these were not possible to separate. Of the remaining models, Model 1c) G-factor plus three correlated domains (i.e. Well-being and Problems merged into one factor) and Model 3c) G-factor plus positive responding and Risk, showed the best fit to data. However, Model 1c) showed a problematic pattern of loadings, with the combined Well-being/Problems factor having no statistically significant loadings and the Functioning factor having both positive and negative loadings. Model 3c) showed adequate loadings for both the G-factor and the specific Risk and Positive responding factors. Still, none of the models fit well according to conventional standards (i.e. significant Chi-square test, RMSEA above .05, and CFI below .90). For this reason, a decision was made to also do an EFA to see whether an alternative structure would emerge for the Kenyan sample.

**Table 1:** Model fit information for Confirmatory Factor Analyses of the Clinical Outcomes in Routine Evaluation - Outcome Measure

Model	X^2^ (df)	p	AIC	RMSEA (95%CI)	CFI	SRMR
1. a) G + four uncorrelated domains	1330.28 (497)	<.001	36287.00	.07 (.06, .07)	.84	.06
1. b) G + four correlated domains^a^	1139.73 (487)	<.001	36116.45	.06 (.06, .07)	.87	.05
1. c) G + three correlated domains^b^	1143.24 (490)	<.001	36113.96	.06 (.06, .07)	.87	.05
2. G + risk	1439.30 (521)	<.001	36348.02	.07 (.07, .08)	.82	.06
3. a) G + pos. responding + four correlated domains^a^	977.80 (482)	<.001	35964.51	.06 (.05, .06)	.90	.04
3. b) G + pos. responding + three correlated domains^b^			No convergence			
4. G + pos. responding + risk	1224.05 (520)	<.001	36134.76	.06 (.06, .07)	.86	.05

^a^The correlation between Well-being and Problems was estimated as > 1.0 in all models with four correlated domains

^b^In the models with three correlated domains, Well-being and Problems were merged

### Exploratory factor analysis

Exploratory Factor Analysis was run using Maximum Likelihood estimation. Scree plot analysis indicated either 3- or 4 factors. Parallel analysis [18] suggested a 4-factor solution, although the fourth eigenvalue was only marginally larger (.03) for the observed covariance matrix than the average eigenvalue for the simulated data. Thus, 3- and 4-factor solutions were explored in terms of interpretability and factor structure. Two different rotation methods were tested, first oblique rotation and then bifactor rotation. Output for the bifactor rotation method seemed more interpretable, so this method was chosen. Both 3- and 4- factor models had a strong G-factor, a factor for the Risk items, and a factor for the Positively framed items. The fourth factor in the 4-factor solution was hard to interpret and its highest loading was .38, so the 3-factor solution was chosen. Loadings for all items on the three factors are presented in Table 2. As can be seen, the pattern fits well with the G-factor, Risk items, and Positively framed items. This structure is highly similar to the factor structure found for English language CORE-OM with data from the UK [7]. However, it should be noted that model fit indices for this model (χ^2^ (462) = 1100.97, RMSEA = .06 (95% CI .06, .07), CFI = .87, SRMR = .04) did still not quite match conventional standards for model fit of SEM models, at least not the CFI which should be >.90 according to most sources (e.g. [19]).

**Table 2:** Exploratory Factor Analysis of the Clinical Outcomes in Routine Evaluation - Outcome Measure with Bifactor Rotation

Item	G-factor	Risk	Positive responding
I have felt terribly alone and isolated	0.63*	0.06	-0.20*
I have felt tense, anxious or nervous	0.62*	-0.03	-0.31*
I have felt I have someone to turn to for support when needed	0.28*	0.02	0.32*
I have felt O.K. about myself	0.56*	-0.06	0.27*
I have felt totally lacking in energy and enthusiasm	0.56*	0.01	-0.09
I have been physically violent to others	0.44*	0.50*	0.04
I have felt able to cope when things go wrong	0.47*	0.04	0.30*
I have been troubled by aches, pains or other physical problems	0.41*	0.11	-0.24*
I have thought of hurting myself	0.58*	0.54*	0.01
Talking to people has felt too much for me	0.33*	0.09	-0.03
Tension and anxiety have prevented me doing important things	0.61*	-0.03	-0.14*
I have been happy with the things I have done	0.59*	0.00	0.34*
I have been disturbed by unwanted thoughts and feelings	0.70*	0.00	-0.01
I have felt like crying	0.64*	0.16*	-0.00
I have felt panic or terror	0.65*	0.08	-0.19*
I made plans to end my life	0.53*	0.44*	-0.01
I have felt overwhelmed by my problems	0.74*	-0.06	-0.15*
I have had difficulty getting to sleep or staying asleep	0.61*	-0.02	-0.22*
I have felt warmth or affection for someone	0.26*	0.04	0.37*
My problems have been impossible to put to one side	0.67*	-0.09	-0.02
I have been able to do most things I needed to	0.46*	-0.06	0.40*
I have threatened or intimidated another person	0.41*	0.34*	-0.05
I have felt despairing or hopeless	0.75*	0.07	0.02
I have thought it would be better if I were dead	0.69*	0.37*	-0.00
I have felt criticised by other people	0.62*	0.09	-0.01
I have thought I have no friends	0.64*	0.09	-0.05
I have felt unhappy	0.80*	-0.06	0.02
Unwanted images or memories have been distressing me	0.65*	-0.03	-0.01
I have been irritable when with other people	0.72*	0.05	0.02
I have thought I am to blame for my problems and difficulties	0.36*	0.05	-0.07
I have felt optimistic about my future	0.28*	0.03	0.45*
I have achieved the things I wanted to	0.54*	-0.25*	0.35*
I have felt humiliated or shamed by other people	0.65*	-0.04	0.07
I have hurt myself physically or taken dangerous risks with my health	0.53*	0.37*	0.03

Items with loadings ≥ .32 in bold text [16]

*p < .05

### Unidimensionality of the 28 non-risk items?

From the results so far, it seems fairly clear that the risk items - although strongly related to the general distress factor, might be usefully treated as a separate index since they apparently include important information that is not included in the general distress factor. It is less clear what to do about the eight positively framed items. Using the loadings from Table 2, the ECV was calculated as .81, meaning that 81% of variance across all 34 items of the CORE-OM can be explained by the general factor. If the risk items are removed, the ECV goes up to .86. These are both high scores, suggesting that the lion’s share of the variance in the CORE-OM items is due to the general distress factor.

### Reliability of the general distress factor

An additional useful statistic is the Coefficient Omega Hierarchical, which is a measure of reliability of the general factor in a bifactor model. This is calculated as the square of the sum of loadings on the general factor divided by (the square of the sum of loadings on the general factor plus the sum of the square of loadings of specific factors and the sum of residual variances). The coefficient Omega Hierarchical was calculated as .92 across all 34 items. This means that using the sum or mean of all 34 items will result in a reliable measure of the general distress factor despite the fact that variance due to risk and positive responding will be treated as error variance. If the risk items were removed, Omega Hierarchical increased marginally (to .93). Removing also positively framed items did not affect Omega Hierarchical further.

To check the reliability of the risk subscale, we also calculated Omega Hierarchical for an index of these six items. The reliability of this index was only .33 for risk uninfluenced by the general distress factor. However, it does not seem reasonable to remove general distress from the risk scale, and if the general factor was retained within the risk factor the reliability was .84.

## Discussion

The CORE-OM has been translated into several languages and has yielded slightly different factor structures in different samples. Results of the present study indicate that the English version of the CORE-OM was acceptable to patients attending hospital based psychiatric care in urban Nairobi. Given that a meaningful factor structure emerged it also seemed to have been understandable, although this was not tested directly. This is an important, positive finding for cross-cultural application of CORE-OM, given possible language and cultural barriers around expression of idioms of distress, and functional literacy problems in the population visiting public hospitals in Nairobi.

The factor structure for the Kenyan version of the CORE-OM was highly similar to the one found in British data [7], with a strong general distress factor plus additional factors for risk items and positively framed items. A difference was that in our sample we were unable to find any meaningful differentiation between the original CORE-OM domains, especially not between well-being and psychological problems. However, although the British factor analysis showed better model fit for a model including the four CORE-OM domains than for the model with only general distress, method factors and risk, the non-risk domain factors in their study explained very little variance (well-being 1%, psychological problems 6%, and functioning 8%; compared to 39% for the risk factor). The factor analysis [8] on the Norwegian version of the CORE-OM found the model with a general factor plus the four domains to fit the data better than a model without the problems, well-being and functioning domains. In that study, the pattern of loadings can be said to provide good support for the risk factor (22% explained variance) reasonable support for the psychological problems domain (12% explained variance), while well-being and psychological problems explaining little variance (1% and 5%, respectively). Both Norwegian and British factor analyses [7, 8] found quite similar amounts of variance explained for the general distress factor (32% and 29%) as we did (33%).

The practical implication of this is that it seems to be possible to use the sum or mean of all 34 CORE-OM items as a reliable measure of general psychological distress in a Kenyan population. The bias due to differential responding to positive items seems to be negligible, since reliability was excellent even if the variance due to positive responding was treated as error variance. If risk of violence to self- and/or others is an important factor to be studied, it also seems possible to create a separate reliable index of the six risk items, while keeping in mind that the risk items are substantially affected by the general distress factor.

Strengths of this study include the wide age group to which CORE-OM was administered as well as the mostly lower-class population studied. This would be one of the first studies in Kenya to study a comprehensive self-report measure that assesses psychological distress rather than psychiatric interview schedules that tend to focus on discrete symptoms rather than continuous distress and well-being. It is certainly one of the few studies that will potentially build greater evidence towards consolidating a psychological understanding of mental illnesses in Kenya.

There are some limitations of the present study: First, we only tested the factor structure at a single time-point. This means that we cannot determine whether the CORE-OM works as a measure of change in these settings. Specifically, longitudinal factor invariance, test-retest reliability, and sensitivity to change will all need to be evaluated before the measure can confidently be used as an outcome measure in these contexts. In addition, our design in the present study did not enable us to test the possibility that the CORE-OM misses important types of distress that are important for Kenyans seeking mental health care. This issue is, however, partly addressed by other studies from our research group (e.g. [20]).

The minimum sample size needed for factor analysis is a source of confusion, with common recommendations having little empirical support [21]. Minimum sample size depends on the size of communalities (i.e. variance in indicator variables explained by the factors, which should be large) and the number of variables per factor (the more variables per factor the better). In our case we had fairly low communalities (many below .5), but also many variables per factor (on average more than 10). According to the simulations reported in [21], this would – in combination with our sample size (*N* = 345) yield excellent recovery of the population factor structure (congruence around .98). In addition, our results were consistent with a structure found in prior research [7]. Still, replication in a larger sample would be desirable.

Another limitation is that model fit according to the CFI was below conventional standards even for the best fitting models. Still, it is surprising that the CFI showed inadequate fit when other indices such as the SRMR and RMSEA were if not excellent so at least adequate. Since the CFI compares model fit to the fit of an independence model (i.e. a model assuming zero correlations among all items), it is possible for the CFI to be low when correlations between items are, on average, low (which means that the independence model will fit relatively good). It has been suggested [22] that when the RMSEA of the independence model is below .158, the CFI should not be calculated since it will be negatively biased. In the present data, the RMSEA of the independence model was .162, i.e. very close to this cut-off. So, it seems likely that the low CFI was due to a too well-fitting independence model.

## Conclusions

The English language version of the CORE-OM was shown to be acceptable to patients and with similar factor structure in a sample of mostly lower-class patients seeking treatment at psychiatric clinics in Nairobi. The measure captures general psychological distress reliably, and can also be used to measure risk for harm to self- and/or others.

## Abbreviations

CFI: Confirmatory Factor Analysis
CORE-OM: Clinical Outcomes in Routine Evaluation – Outcome Measure
EFA: Exploratory Factor Analysis
KNH: Kenyatta National Hospital
MNH: Mathare National Hospital
RMSEA: Root Mean Square Error of Approximation
SEM: Structural Equation Modelling
SRMR: Standardized Root Mean Square Residual
UoN: University of Nairobi

## Footnotes

1 The CORE instruments are free to reproduce without fee both on paper and in software but that they are all copyright to CORE System Trust (https://www.coresystemtrust.org.uk/home/copyright-licensing/).

2 The CORE System Trust has a webpage devoted to translations, see https://www.coresystemtrust.org.uk/translations/?
